# Identification of Sex Differentiation-Related microRNAs in Spinach Female and Male Flower

**DOI:** 10.3390/ijms23084090

**Published:** 2022-04-07

**Authors:** Ning Li, Yueyuan Wang, Jiwen Wang, Wanqing Zhang, Ziwei Meng, Yuanshen Wang, Yulan Zhang, Shufen Li, Wujun Gao, Chuanliang Deng

**Affiliations:** College of Life Sciences, Henan Normal University, Xinxiang 453007, China; lining2310@163.com (N.L.); wangyueyuan0407@163.com (Y.W.); wjw960908@163.com (J.W.); zwq5921@163.com (W.Z.); mengziwei2022@163.com (Z.M.); wys139816@163.com (Y.W.); 2019056@htu.edu.cn (Y.Z.); lishufen@htu.edu.cn (S.L.); gaowujun@htu.cn (W.G.)

**Keywords:** microRNA, sex determination and differentiation, spinach, unisexual flower, dioecism

## Abstract

Sex determination and differentiation is an important biological process for unisexual flower development. Spinach is a model plant to study the mechanism of sex determination and differentiation of dioecious plant. Till now, little is known about spinach sex determination and differentiation mechanism. MicroRNAs are key factors in flower development. Herein, small RNA sequencing was performed to explore the roles of microRNAs in spinach sex determination and differentiation. As a result, 92 known and 3402 novel microRNAs were identified in 18 spinach female and male flower samples. 74 differentially expressed microRNAs were identified between female and male flowers, including 20 female-biased and 48 male-biased expression microRNAs. Target prediction identified 22 sex-biased microRNA-target pairs, which may be involved in spinach sex determination or differentiation. Among the differentially expressed microRNAs between FNS and M03, 55 microRNAs were found to reside in sex chromosome; one of them, *sol-miR2550n*, was functionally studied via genetic transformation. Silencing of *sol-miR2550n* resulted in abnormal anther while overexpression of *sol-miR2550n* induced early flowering, indicating *sol-miR2550n* was a male-promoting factor and validating the reliability of our small RNA sequencing data. Conclusively, this work can supply valuable information for exploring spinach sex determination and differentiation and provide a new insight in studying unisexual flower development.

## 1. Introduction

Sex is a special character of biology. Most animals are unisexual and the mechanism of sex determination mammalian is already clearer. In plant kingdom, only 6% of angiosperms are dioecious plants and their sex determining mechanism are not well studied [1]. The gender origins of dioecious plant are not uniform, which enhances the difficulty to uncover plant sex determining mechanism. At present, there are two main ideas about the mechanism of sex determination in dioecious plants: one is the two-gene mutation model, and the other is the single-gene mutation model. For two-gene mutation model, the appearance of dioecious plants may be due to the mutation of two genes, *M* gene, which controls stamen fertility, and *F* gene, which regulates pistil development [2,3]. For single-gene mutation model, the emergence of dioecious plants may be caused by the two independent mutations of the same gene *Q*: one is loss-of-function mutation resulting in male sterility, and the other is gain-of-function mutation inducing female sterility [4,5,6]. From these two models, it can be seen that the mutation of flower development-related genes is the most important factor leading to the generation of plant sex. To date, the sex-determining genes or candidates have been identified in a few angiosperms such as *Vitis vinifera* [7,8], *Diospyros lotus* [9], *Populus trichocarpa* [10,11,12], *Ficus carica* [13], *Date palm* [14], *Fragaria octoploids* [15], *Actinidia chinensis* [16] and *Asparagus officinalls* [17]. The function of the sex-determining genes was studied only in few plants and the mechanism of sex determination remains largely elusive. Sex differentiation is an important process along with sex determination. Researches on sex differentiation may supply clues to uncover sex determination. Many genes related to sex differentiation have been identified in different plants such as in *Silene latifolia* [18,19], *Carica papaya* [20].

MicroRNAs (miRNAs) are a class of endogenous single-stranded non-coding small RNA molecules with a length of about 21–24 nt. The initial transcription product pri-miRNA is spliced into a single strand RNA precursor (pre-miRNA) with hairpin structure, and then formed the mature miRNA with regulatory function. MiRNAs negatively regulate their target messenger RNAs (mRNA) through mRNA degradation, translation inhibition or chromatin modification [21,22,23]. MiRNAs play important regulatory roles in plant morphogenesis, nutrient balance, biological and abiotic stress [24,25,26,27]. More and more studies have shown that miRNAs played important roles in plant flower development. Flowering is an important process in plant growth cycle and miRNAs are involved in flower formation processes. In *Arabidopsis*, miR164c regulated two NAC transcription factors *CUP-SHAPED COTYLEDON1* (*CUC1*) and *CUC2* to control the boundary foundation of floral organs [28]. MiR172 can regulate the formation of sepal and petal primordium via inhibiting the translation of floral homeotic gene *APETALA2* [29,30]. MiR159, miR167 and miR319 can form a regulation module to influence flower development by targeting transcription factors *MYB33*, *TCP4* and *Auxin Response Factor6/8* [31]. In tomato, sly-miR160a was involved in flower development via regulating auxin response factor *SlARF10A* [32]. The flowers of corn, a hermaphrodite plant, are bisexual in the early development stage but become unisexual in the late stage as the development of pistil or stamen is blocked. It has been reported that zma-miR172E was a member of miR172 family and an insertional mutation in the promoter region of its coding gene resulted in a corn recessive mutant *tasseleseed4*, in which carpel developed in tassel with no stamen [33]. The sex determining gene of a dioecious plant, persimmon (*Diospyros lotus*) was identified as a miRNA, *OGI*, targeting a homeodomain transcription factor *MeGI* to determine plant sex [9,34]. These studies fully demonstrate that miRNAs participate in flower development, even in the formation of unisexual flowers and moreover miRNA can serve as sex determining gene.

*Spinacia oleracea* (2n = 12), a dioecious plant, has a pair of homomorphic sex chromosomes (XY) and is at the early stage of sex chromosome evolution [35]. Many sex-linked, sex chromosome-specific and sex-determining-gene-linked molecular markers were developed in spinach, such as T11A, V20A, 45 s rDNA, SP_0018, SpoX and so on [36,37,38,39]. These molecular markers are helpful for locating the accurate sex-determining region (SDR) and further identifying sex-determining genes. Qian et al. (2017) [40], Yu et al. (2021) [41] and Ma et al. (2022) [42] found the SDR locating on the sex chromosome using different method. Moreover, 166 sex-differentiation-related genes and 12 Y-specific genes were successively reported in spinach [40,43]. However, the function of these genes was still unclear and it is still controversial that spinach sex determination is regulated by single-gene mutation model or two-gene mutation model [43]. Spinach genome was firstly published in 2017 and constantly updated with the development of high throughput technology, which supply valuable information for functional genome study [41,42,44,45,46].

To verify whether miRNA can serve as sex determining gene in spinach, miRNAs resource should be firstly harvested. In 2017, spinach miRNAs were analyzed via in silico prediction in vegetative tissues of spinach [47]. Flower development-related genes are crucial for sex determination and differentiation, so we performed small RNA sequencing using 18 spinach flower samples at three early female and male flower development stages to identify some candidate miRNAs for spinach sex determination or differentiation. Virus-induced gene silencing (VIGS) technology is an efficient way to study gene function in plant [48]. In addition, this technology has been successfully applied in spinach [49,50]. Moreover, RNA interference technology and overexpression technology have been used to study miRNA function [51]. Hence, we performed VIGS and overexpression technology to analyze the function of *sol-miR2550n*, one of the candidate miRNAs, and to further validate the accuracy of our sequencing data.

## 2. Results

### 2.1. Small RNA Sequencing

**Figure 1:**
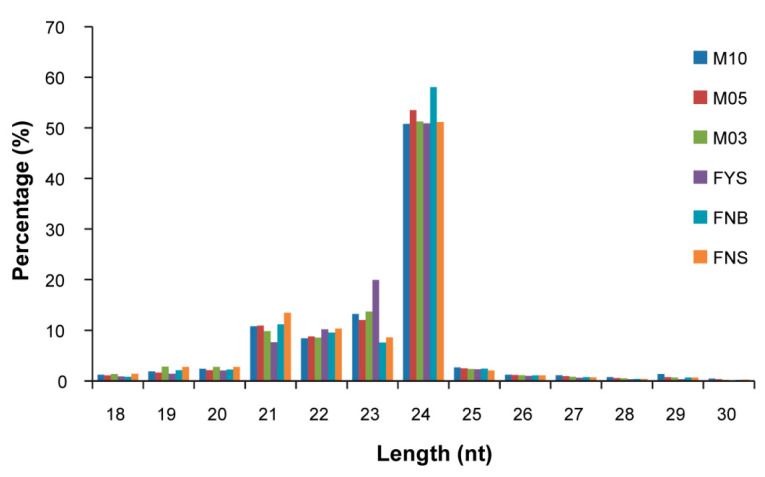
Frequency distribution of small RNA length. FNS, female flower without stigma (0.3 mm in diameter); FNB, female flower without stigma (0.5 mm in diameter); FYS, mature female flower with stigma; M03, male flower (0.3 mm in diameter); M05, male flower (0.5 mm in diameter); M10, male flower (1.0 mm in diameter).

The roles of miRNAs in flower development are not clear in spinach till now. To characterize miRNAs in spinach, we performed small RNA sequencing using 18 spinach flower samples and harvested 13,911,586 high quality reads (mean value of 18 samples data) (Table 1). After filtering, at least 91.37% of high quality reads were selected as small RNA clean tags, the primary length distribution of which is 24 nt (Figure 1). All small RNA clean tags were aligned in GeneBank database (Release 209.0), Rfam database (11.0), spinach genome (http://www.spinachbase.org/, accessed on 23 July 2021) and miRBase database (Release 21). Finally, 92 known miRNAs and 3402 novel miRNAs were identified in 18 spinach flower samples (Table 2).

### 2.2. Sex-Biased miRNAs of Spinach

**Figure 2:**
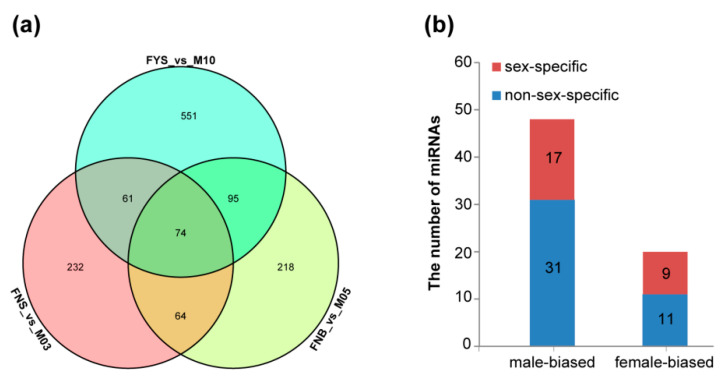
Differentially expressed miRNAs. (**a**) the Venn diagram of DE miRNAs among three flower stages; (**b**) the number of sex-biased expression miRNAs.

According to the expression of miRNA, differentially expressed miRNAs (DE miRNAs) were analyzed among female and male flower samples. There were 431 DE miRNAs between FNS and M03, 451 DE miRNAs between FNB and M05, and 781 DE miRNAs between FYS and M10 (Figure 2a). Sex-biased genes, which exhibit significantly higher expression in one sex than in the other sex, always act downstream of sex-determining gene. Hence, identification of the sex-biased genes is helpful to uncover the sex-determination mechanism. Herein, 74 DE miRNAs (7 were known and 67 were novel) were identified between female and male flowers at three early developmental stages (Figure 2a; Table S1); and of them, 20 miRNAs displayed female-biased expression and 48 miRNAs displayed male-biased expression (Figure 2b). Moreover, there were 9 female-specific expression miRNAs and 17 male-specific expression miRNAs (Figure 2b). These sex-biased and sex-specific expression miRNAs may be involved in spinach sex determination or differentiation.

### 2.3. Target Genes of miRNAs

**Figure 3:**
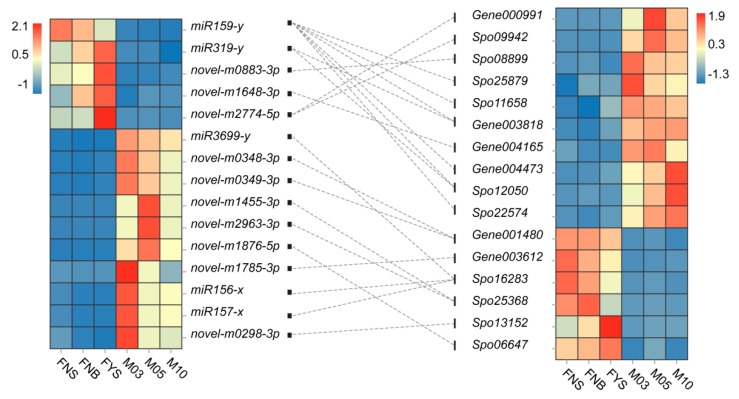
22 pairs of sex-biased miRNA-target. The heatmaps represent the expression profile of 15 sex-biased miRNAs (**left**) and 16 sex-biased target genes (**right**); the dotted line links miRNA and its target.

Target genes of miRNAs were analyzed according to our previous transcriptome data [52]. Totally, 20,460 genes were predicted as targets of 2855 miRNAs and 49,591 target sites were found in these target genes for miRNAs binding (Table 3). It can be seen that one miRNA can regulate more than one gene and one gene can be controlled by more than one miRNA. MiRNA and its target always display antagonistic expression profile. As previously reported [52], 1946 male-biased genes and 961 female-biased genes were found between female and male flowers at three early developmental stages. Herein, we found ten male-biased genes were targeted by five female-biased miRNAs and six female-biased genes were targeted by ten male-biased miRNAs, composing 22 miRNA-target pairs (Figure 3, Table S2). The 22 miRNA-target pairs may be involved in spinach sex determination or differentiation.

### 2.4. MiRNAs Residing in Sex Chromosome

**Figure 4:**
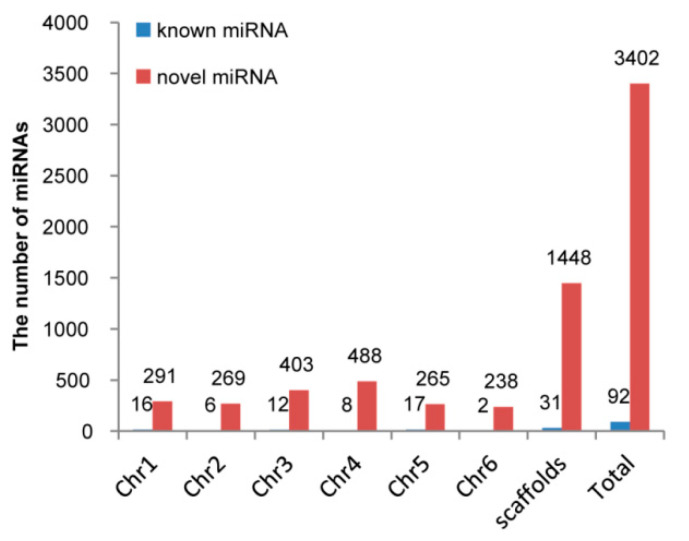
Distribution of known miRNAs and novel miRNAs in spinach chromosomes.

In the published spinach genome data, chr4 is the sex chromosome where the sex-determining genes reside [43]. Blasting with spinach genome [44], we found 307 miRNAs (16 known and 291 novel) residing in Chr1, 275 miRNAs (6 known and 269 novel) residing in Chr2, 415 miRNAs (12 known and 403 novel) residing in Chr3, 496 miRNAs (8 known and 488 novel) residing in Chr4 (sex chromosome), 282 miRNAs (17 known and 265 novel) residing in Chr5, 240 miRNAs (2 known and 238 novel) residing in Chr6, and 1479 miRNAs (31 known and 1448 novel) residing in scaffolds (Figure 4). Among 496 miRNAs residing in sex chromosome, there were 55 DE miRNAs between FNS and M03, 56 DE miRNAs between FNB and M05, and 114 DE miRNAs between FYS and M10. However, no intersection was existed among these three data sets, i.e., no DE miRNA was identified between female and male flowers at three developmental stages. Considering that sex determination and differentiation occurs at the earlier flower development stage in spinach, so we further analyzed DE miRNAs between FNS and M03, the earliest stage of the three flower developmental stages. As a result, 14 differential expression genes were found as targets of ten DE miRNAs between FNS and M03 (Table 4).

### 2.5. Functional Analysis of Sol-miR172 and Sol-miR2550n

**Figure 5:**
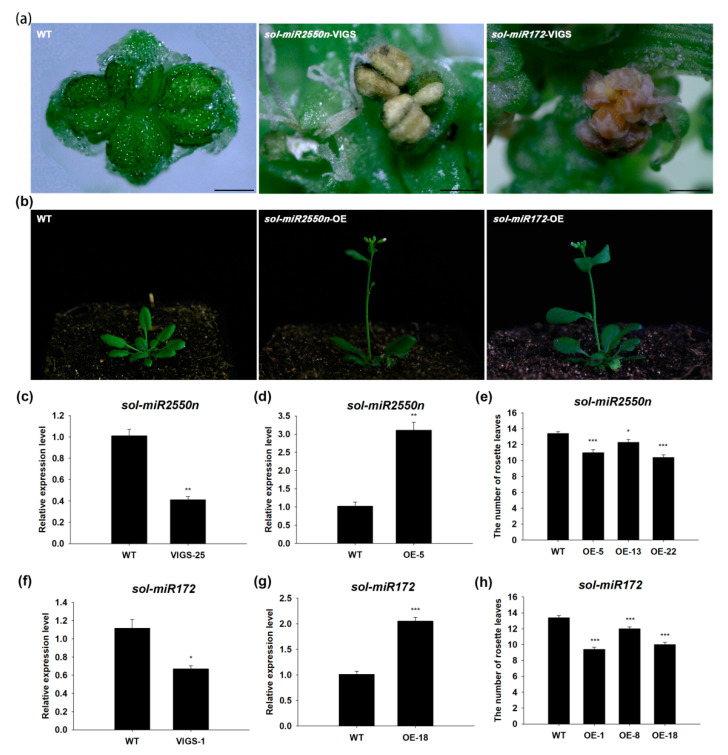
Phenotypes of silencing and overexpression of *sol-miR2550n* and *sol-miR172*. (**a**) anther phenotype of wildtype (WT), *sol-miR2550n-* or *sol-miR172*-silencing spinach male plant, scale bar = 0.5 mm; (**b**) phenotype of wildtype, *sol-miR2550n-* or *sol-miR172*-overexpressed *Arabidopsis* plant; (**c**) relative expression level of *sol-miR2550n* in wildtype and *sol-miR2550n*-silencing spinach plant; (**d**) relative expression level of *sol-miR2550n* in wildtype and *sol-miR2550n*-overexpressed *Arabidopsis* plant; (**e**) statistics of flowering time of *sol-miR2550n-*overexpressed *Arabidopsis* plants, n ≥ 20; (**f**) relative expression level of *sol-miR172* in wildtype and *sol-miR172-*silencingspianch plant; (**g**) relative expression level of *sol-miR172* in wildtype and *sol-miR172*-overexpressed *Arabidopsis* plant; (**h**) statistics of flowering time of *sol-miR172*-overexpressed *Arabidopsis* plants, n ≥ 20; “*” represents *p*-value < 0.05; “**” represents *p*-value < 0.01; “***” represents *p*-value < 0.001; error bar represents standard error.

To uncover the potential function of these miRNAs residing in sex chromosome, we performed plant transformation experiments for the first time. *sol-miR172* (*miR172-y*) is a DE miRNA between FNS and M03 and showed higher expression level in M03 stage; its homolog in *Arabidopsis* has been reported to regulate flower development [29], so we selected *sol-miR172* as a positive control. *sol-miR2550n* (*novel-m2550-5p*) resided in sex chromosome and displayed higher expression level in M03 stage than in FNS stage (Table S3), so we selected it as a sex-determining candidate. Virus-induced-gene-silencing and heterologous-overexpression were performed for functional analysis of *sol-miR172* and *sol-miR2550n* in spinach. Silencing of *sol-miR172* or *sol-miR2550n* in spinach resulted in abnormal male flower (anther abortion) (Figure 5a,c,f). Overexpression of *sol-miR172* or *sol-miR2550n* in *Arabidopsis* induced early flowering (Figure 5b,d,e,g,h). The phenotype of overexpression *sol-miR172* in *Arabidopsis* was consistent with Aukerman and Sakai’s report [53]. These results verified the accuracy of the small RNA sequencing and analysis. Moreover, this work will supply abundant valuable information to explore the mechanism of spinach sex determination and differentiation.

## 3. Discussion

Sex determination and differentiation is an important question for monoecious and dioecious plant. In some species, the sex determining genes have been identified, such as asparagus, kiwifruit and persimmons. However, sex determining gene is still unclear in spinach; it may be transcription factor or noncoding RNA. Hence, we performed small RNA sequencing to search some clues.

It was the first time to sequencing small RNAs in spinach. Totally, 92 known miRNAs and 3402 novel miRNAs were identified in 18 spinach flower samples (Table 1), which enriched plant miRNA resource. To analyze the miRNAs related to spinach sex determination or differentiation, 74 differentially expressed miRNAs were screened out among female and male flower at three early developmental stages (Figure 2a), including 20 female-biased miRNAs, 9 female-specific miRNAs, 48 male-biased miRNAs and 17 male-specific expression miRNAs (Figure 2b). Moreover, 22 miRNA-target pairs were found through target prediction (Figure 3). These 74 sex-biased or sex-specific miRNAs can be served as candidates of spinach sex differentiation, but not sex determination as they were not reside in sex chromosome. Genes residing in sex chromosome, especially in sex-determining region, are important clues to explore spinach sex determination and differentiation. Hence, we analyzed the miRNAs residing in sex chromosome; and then we found 496 miRNAs in sex chromosome, but none was sex-biased miRNA (Figure 4). Considering that sex determinant plays a role in early spinach flower development stage, so we screened out ten DE miRNAs between FNS and M03, two earliest stages among samples (Table 4). 14 targets with opposite expression trend to these ten DE miRNAs were also identified (Table 4). The function of these ten DE miRNAs residing in sex chromosome will be studied in our future work.

Herein, one of the DE miRNAs residing in sex chromosome, *sol-miR2550n*, were firstly studied for its function. Through VIGS and overexpression method, we found that silencing of *sol-miR2550n* in spinach induced abnormal male flower (anther abortion) and overexpression of *sol-miR2550n* in *Arabidopsis* resulted in early flowering (Figure 5). *sol-miR2550n* showed higher expression level in male flower (M03) than in female flower (FNS) (Table S3), hence down-regulation of its expression influenced the male flower development. However, up-regulation of its expression didn’t affect flower structure but induced early flowering. In spinach, the flowering time of male plant is earlier than female plant [54]. Hence, an interesting hypothesis is that up-regulation of a male factor may promote the male trait, such as early flowering. Hence, *sol-miR2550n* may be a male-promoting factor. The exact molecular mechanism needs to be explored in future work. Meanwhile, *sol-miR172*, a homolog of the well-known flower-related factor *miR172* [53,55], was also studied in this work as a positive control. In spinach, *sol-miR172* also showed higher expression level in M03 (male flower) than in FNS (female flower) but not resided in sex chromosome. Overexpression of *sol-miR172* in *Arabidopsis* resulted in early flowering, in accordance with *ath-miR172* (Figure 5). However, down-regulation of *sol-miR172* in spinach resulted in anther abortion of male flower, which is not similar with *ath-miR172* (influencing flower whorls pattern) (Figure 5). Such difference may be due to the architecture difference between unisexual flower and bisexual flower and also indicated a new potential regulation pathway of *miR172* in unisexual flower development. The molecular mechanism will be studied in our future work.

## 4. Materials and Methods

### 4.1. Plant Materials and Growth Conditions

All plants used in this study were *Spinacia oleracea* L. cv DA JIAN YE BO CAI, a dioecious plant. Seeds were obtained from U.S. National Plant Germplasm System (https://npgsweb.ars-grin.gov/gringlobal/search.aspx?, accessed on 23 July 2021, accession number is PI 527332) and grown in an experimental field at Henan Normal University, Xinxiang, China (113.90° E, 35.32° N). Female and male flowers collected at three stages were separately used for Small RNA Sequencing and qRT-PCR. Flower samples were same with our previous published paper [52].

Spinach plants used for VIGS treatment were planted in climate chamber (Jiangnanyiqi, Ningbo, China) with 16 h/8 h day/night, 18 °C, 60% humidity. *Arabidopsis* plants were planted in climate chamber (Jiangnanyiqi, Ningbo, China) with 16 h/8 h day/night, 22 °C, and 60% humidity.

### 4.2. Library Construction and Sequencing

After total RNA was extracted by TRIzol (Thermo Fisher Scientific, Waltham, MA, USA), the RNA molecules in a size range of 18–30 nt were enriched by polyacrylamide gel electrophoresis (CWBIO, Taizhou, China). Then the 3′ adapters were added and the 36–44 nt RNAs were enriched. The 5′ adapters were then ligated to the RNAs as well. The ligation products were reverse transcribed by PCR amplification and the 140–160 bp size PCR products were enriched to generate a cDNA library and sequenced using Illumina HiSeq TM 2500 (Illumina, San Diego, CA, USA) by Gene Denovo Biotechnology Co. (Guangzhou, China).

Reads obtained from the sequencing machine included dirty reads containing adapters or low quality bases which would affect the following assembly and analysis. Thus, to acquire clean tags, raw reads were further filtered using fastp software (https://github.com/OpenGene/fastp, version 0.12.4, 1 March 2022) according to the following rules:

(1) Removing low quality reads containing more than one low quality (Q-value ≤ 20) base or containing unknown nucleotides (N);
(2) Removing reads without 3′adapters;
(3) Removing reads containing 5′adapters;
(4) Removing reads containing 3′ and 5′ adapters but no small RNA fragment between them;
(5) Removing reads containing ployA (the content of A base in a reads is higher than 70%) in small RNA fragment;
(6) Removing reads shorter than 18 nt (not include adapters).

### 4.3. Alignment and Identification of Small RNA

All of the clean tags were aligned with small RNAs in GeneBank database (https://www.ncbi.nlm.nih.gov/genbank/, Release 209.0, 1 March 2022) using blast (https://blast.ncbi.nlm.nih.gov/Blast.cgi, version 2.2.25, 1 March 2022) (blastn, identity > 97%) to identify and remove rRNA, scRNA, snoRNA, snRNA and tRNA. Meanwhile all of the clean tags were aligned with small RNAs in Rfam database (http://rfam.xfam.org/, version 11.0, 1 March 2022) using blast (https://blast.ncbi.nlm.nih.gov/Blast.cgi, version 2.2.25, 1 March 2022) (blastn, identity > 97%) to identify and remove rRNA, scRNA, snoRNA, snRNA and tRNA. All of the clean tags were also aligned with reference genome [44] using bowtie (https://www.nature.com/articles/nmeth.1923, version 1.1.2, 1 March 2022) (parameters: -v 0 --best --strata –a). Those mapped to exons or introns might be fragments from mRNA degradation, so these tags were removed. The tags mapped to repeat sequences using RepeatMasker (http://www.repeatmasker.org/, 1 March 2022, version open-4.0.6, RepeatMasker Database: RepeatMaskerLib.embl Update 20150807, parameters: RepeatMasker -engine wublast -s -no_is -cutoff 255 -frag 20000) were also removed.

After removing rRNA, scRNA, snoRNA, snRNA, tRNA, mRNA degradation fragments and repeat sequences, the clean tags were then searched against miRBase database (http://www.mirbase.org/, 1 March 2022, Release 21) using bowtie (http://bowtie-bio.sourceforge.net/index.shtml, 1 March 2022, version 1.1.2, parameters: -v 0 --best --strata -a) to identify exist and known miRNAs. Then the unidentified clean tags were mapped to reference genome [44]. According to their genome positions and hairpin structures predicted by software Mireap_v0.2 (https://sourceforge.net/projects/mireap/, 1 March 2022), the novel miRNA candidates were identified. The default parameters of software Mireap_v0.2 were as follows:

(1) Minimal miRNA sequence length is 18 nt;
(2) Maximal miRNA sequence length is 25 nt;
(3) Minimal miRNA reference sequence length is 20 nt;
(4) Maximal miRNA reference sequence length is 23 nt;
(5) Maximal copy number of miRNAs on reference is 20;
(6) Maximal free energy allowed for a miRNA precursor is 18 kcal/mol;
(7) Maximal space between miRNA and miRNA* is 300 nt;
(8) Minimal space between miRNA and miRNA* is 16 nt;
(9) Maximal bulge between miRNA and miRNA* is 4 nt;
(10) Maximal asymmetry of miRNA/miRNA* duplex is 4 nt;
(11) Flank sequence length of miRNA precursor is 20 nt.

### 4.4. MiRNA Expression Profiles

Total miRNAs consist of existing miRNAs, known miRNAs and novel miRNAs, based on their expression in each sample, the miRNA expression level was calculated and normalized to transcripts per million (TPM). The formula is as follows: TPM = Actual miRNA counts/Total counts of clean tags*10^6^.

To identify DE miRNAs across samples or groups the formula was shown as follows:


$$\begin{array}{cc} p(x|y)={\left(\frac{N_{2}}{N_{1}}\right)}^{y}\frac{\left(x+y\right)!}{x!y!{\left(1+\frac{N_{2}}{N_{1}}\right)}^{\left(x+y+1\right)}} & \begin{array}{c} C(y\leq y_{min}|x)=\overset{y\leq y_{min}}{\underset{v=0}{\sum }}p(y|x)\\ D(y\geq y_{max}|x)=\overset{\infty }{\underset{y\geq max}{\sum }}p(y|x) \end{array} \end{array}$$

We identified miRNAs with a fold change ≥ 2 and *p* value < 0.05 in a comparison as significant DE miRNAs.

### 4.5. Target Gene Prediction

Based on the sequences of the exist miRNAs, known miRNAs and novel miRNAs, the candidate target genes were predicted. The software patmatch (ftp://ftp.arabidopsis.org/home/tair/Software/Patmatch/, 1 March 2022, version 1.2) was used to predict target genes. The default parameters were as follows:

(1) No more than four mismatches between sRNA & target (G-U bases count as 0.5 mismatches)
(2) No more than two adjacent mismatches in the miRNA/target duplex
(3) No adjacent mismatches in in positions 2–12 of the miRNA/target duplex (5’ of miRNA)
(4) No mismatches in positions 10–11 of miRNA/target duplex
(5) No more than 2.5 mismatches in positions 1–12 of the of the miRNA/target duplex (5’ of miRNA)
(6) Minimum free energy (MFE) of the miRNA/target duplex should be > = 74% of the MFE of the miRNA bound to it’s perfect complement.

### 4.6. qRT-PCR

E.Z.N.A.^®^ Plant miRNA Kit (OMEGA, Norcross, GA, USA) and E.Z.N.A.^®^ Plant RNA Kit (OMEGA, Norcross, GA, USA) was used to isolate miRNA and total RNA, separately, according to manufacturer’s introduction. Mir-X miRNA First-Strand Synthesis Kit (TaKaRa, Shiga, Japan) was used to perform reverse transcription of miRNA. PrimeScript™ RT reagent Kit with gDNA Eraser (TaKaRa, Shiga, Japan) was used to synthesis the cDNA of total RNA. Mir-X miRNA qRT-PCR TB Green Kit (TaKaRa, Shiga, Japan) and TB Green ^®^ Premix Ex Taq™ II (Tli RNaseH Plus) (TaKaRa, Shiga, Japan) were used to perform the qRT-PCR of miRNA and mRNA, respectively. qRT-PCR was carried out on LightCycler^®^ 480 System (Roche, ) according to the manufacturer’s instructions. *SpoEF*, *SpoUBQ*, *At5sRNA*, *AtACTIN2* were used as an internal control for normalization. The relative expression level of transcript was calculated with 2^−ΔΔCT^ method. All primers sequences are listed in Table S4.

### 4.7. Vector Construction and Plant Transformation

The pre-miRNA was cloned using Trans-Start^®^ FastPfu Fly DNA Polymerase (TRANSGEN, Beijing, China) and inserted into the pTRV2 vector (VIGS) or pLP100-35s vector (overexpression) with ClonExpressⅡOne Step Cloning Kit (Vazyme, Nanjing, China). These vectors were transformed into *Agrobacterium* competent cells (GV3101, WEIDI, Shanghai, China). Spinach seedlings with four leaves were injected with *Agrobacterium* infection buffer including pTRV2-pre-miRNA vector; flower phenotype was observed after about 30 days. *Arabidopsis* flower buds were dipped in *Agrobacterium* infection buffer including pLP100-35s-pre-miRNA vector; positive T1 plants were used to observe phenotype.

## 5. Conclusions

It was the first time to sequencing small RNAs in spinach. 92 known and 3402 novel miRNAs were identified, which enriched plant miRNA resource. 74 DE miRNAs including male-biased/specific and female-biased/specific expression miRNAs were identified between male and female flowers. Moreover, target prediction identified 22 sex-biased microRNA-target pairs, which may be involved in spinach sex determination or differentiation. Genes residing in sex chromosome, especially in sex-determining region, are very important for sex determination or differentiation. Hence, miRNAs residing in sex chromosome were analyzed and 55 DE microRNAs between FNS and M03 were identified; and one of them, *sol-miR2550n*, was analyzed via genetic transformation. Silencing of *sol-miR2550n* resulted in abnormal anther while overexpression of *sol-miR2550n* induced early flowering, indicating *sol-miR2550n* may be a male-promoting factor. Conclusively, our work can supply valuable information for exploring spinach sex determination and differentiation and provide a new insight in studying unisexual flower development.

## Figures and Tables

**Table 1:** Summary of small RNA sequencing.

Sample ID	Clean Reads	High Quality	3’Adapter Null	Insert Null	5’Adapter Contaminants	Smaller than 18 nt	PolyA	Clean Tags
SpM10-1	14,183,245 (100%)	13,907,336 (98.0547%)	66,715 (0.4797%)	39,362 (0.2830%)	68,350 (0.4915%)	193,229 (1.3894%)	2113 (0.0152%)	13,537,567 (97.3412%)
SpM10-2	14,464,387 (100%)	14,164,634 (97.9276%)	80,069 (0.5653%)	67,309 (0.4752%)	19,248 (0.1359%)	553,683 (3.9089%)	1861 (0.0131%)	13,442,464 (94.9016%)
SpM10-3	14,506,554 (100%)	14,227,721 (98.0779%)	77,086 (0.5418%)	117,860 (0.8284%)	37,183 (0.2613%)	1,254,040 (8.8141%)	1497 (0.0105%)	12,740,055 (89.5439%)
SpM05-1	15,642,311 (100%)	15,300,380 (97.8141%)	34,255 (0.2239%)	129,458 (0.8461%)	29,207 (0.1909%)	778,380 (5.0873%)	2248 (0.0147%)	14,326,832 (93.6371%)
SpM05-2	15,687,586 (100%)	15,366,874 (97.9556%)	39,542 (0.2573%)	104,255 (0.6784%)	42,694 (0.2778%)	514,753 (3.3498%)	1978 (0.0129%)	14,663,652 (95.4238%)
SpM05-3	13,426,285 (100%)	13,140,154 (97.8689%)	60,121 (0.4575%)	96,948 (0.7378%)	23,471 (0.1786%)	566,578 (4.3118%)	1664 (0.0127%)	12,391,372 (94.3016%)
SpM03-1	14,134,390 (100%)	13,839,374 (97.9128%)	65,717 (0.4749%)	87,932 (0.6354%)	25,498 (0.1842%)	619,950 (4.4796%)	2138 (0.0154%)	13,038,139 (94.2105%)
SpM03-2	14,974,564 (100%)	14,661,075 (97.9065%)	51,713 (0.3527%)	49,690 (0.3389%)	27,909 (0.1904%)	716,623 (4.8879%)	2337 (0.0159%)	13,812,803 (94.2141%)
SpM03-3	14,763,485 (100%)	14,459,587 (97.9416%)	59,870 (0.4141%)	102,487 (0.7088%)	25,196 (0.1743%)	1,058,011 (7.3170%)	1825 (0.0126%)	13,212,198 (91.3733%)
SpFYS-1	14,686,982 (100%)	14,367,641 (97.8257%)	42,045 (0.2926%)	57,856 (0.4027%)	18,191 (0.1266%)	388,472 (2.7038%)	1739 (0.0121%)	13,859,338 (96.4622%)
SpFYS-2	16,785,272 (100%)	16,407,458 (97.7491%)	84,234 (0.5134%)	85,457 (0.5208%)	28,469 (0.1735%)	790,536 (4.8182%)	1885 (0.0115%)	15,416,877 (93.9626%)
SpFYS-3	13,411,587 (100%)	13,139,123 (97.9684%)	59,803 (0.4552%)	75,025 (0.5710%)	19,904 (0.1515%)	554,722 (4.2219%)	1697 (0.0129%)	12,427,972 (94.5875%)
SpFNB-1	12,270,405 (100%)	12,010,945 (97.8855%)	38,498 (0.3205%)	53,131 (0.4424%)	14,985 (0.1248%)	314,233 (2.6162%)	1141 (0.0095%)	11,588,957 (96.4866%)
SpFNB-2	13,192,504 (100%)	12,938,214 (98.0725%)	26,417 (0.2042%)	54,149 (0.4185%)	21,619 (0.1671%)	550,952 (4.2583%)	1196 (0.0092%)	12,283,881 (94.9426%)
SpFNB-3	12,539,936 (100%)	12,279,735 (97.9250%)	53,903 (0.4390%)	38,428 (0.3129%)	17,344 (0.1412%)	364,801 (2.9708%)	1132 (0.0092%)	11,804,127 (96.1269%)
SpFNS-1	14,777,105 (100%)	14,461,450 (97.8639%)	44,934 (0.3107%)	58,975 (0.4078%)	19,961 (0.1380%)	822,130 (5.6850%)	1283 (0.0089%)	13,514,167 (93.4496%)
SpFNS-2	13,104,189 (100%)	12,831,211 (97.9169%)	26,074 (0.2032%)	66,202 (0.5159%)	19,029 (0.1483%)	426,303 (3.3224%)	1346 (0.0105%)	12,292,257 (95.7997%)
SpFNS-3	13,170,448 (100%)	12,905,639 (97.9894%)	97,757 (0.7575%)	67,113 (0.5200%)	18,541 (0.1437%)	619,765 (4.8023%)	1122 (0.0087%)	12,101,341 (93.7679%)

**Table 2:** Summary of miRNAs identified in each sample.

Sample	Tags Total	Known miRNA			Novel miRNA		
		miRNA Number	Tags Number	Ratio ^a^	miRNA Number	Tags Number	Ratio ^b^
SpM10-1	13537567	164	655285	4.84%	2434	66103	0.49%
SpM10-2	13442464	179	704922	5.24%	2503	81406	0.61%
SpM10-3	12740055	178	913251	7.17%	2216	78922	0.62%
SpM05-1	14326832	183	694889	4.85%	2416	61441	0.43%
SpM05-2	14663652	166	1005695	6.86%	2338	61939	0.42%
SpM05-3	12391372	173	677457	5.47%	2384	58427	0.47%
SpM03-1	13038139	157	449864	3.45%	2581	95857	0.74%
SpM03-2	13812803	219	662634	4.80%	2563	105093	0.76%
SpM03-3	13212198	194	734663	5.56%	2386	121533	0.92%
SpFYS-1	13859338	173	347553	2.51%	2772	74358	0.54%
SpFYS-2	15416877	200	358872	2.33%	2790	104483	0.68%
SpFYS-3	12427972	163	360486	2.90%	2499	69776	0.56%
SpFNB-1	11588957	140	733948	6.33%	1980	48237	0.42%
SpFNB-2	12283881	155	656934	5.35%	1768	42769	0.35%
SpFNB-3	11804127	131	768099	6.51%	1796	45868	0.39%
SpFNS-1	13514167	163	1010772	7.48%	1857	74367	0.55%
SpFNS-2	12292257	144	977760	7.95%	2107	59193	0.48%
SpFNS-3	12101341	156	687684	5.68%	2032	40874	0.34%
Total ^c^		92			3402		

**Note:**^a^, the ratio of known miRNA Tags number to Tags total; ^b^, the ratio of novel miRNA Tags number to Tags total; ^c^, the total number of known or novel miRNAs identified in all samples.

**Table 3:** Statistics of target gene prediction of all miRNAs.

Sample Name	miRNA Number	Target Gene Number	Target Site Number
SpM10-1	1726	7161	14391
SpM10-2	1796	7619	15036
SpM10-3	1598	7832	15006
SpM05-1	1746	8359	15876
SpM05-2	1679	6518	13201
SpM05-3	1720	7744	15111
SpM03-1	1822	6874	13919
SpM03-2	1893	9451	18542
SpM03-3	1712	9045	17370
SpFYS-1	1952	6646	14188
SpFYS-2	1976	7817	16164
SpFYS-3	1744	6518	13423
SpFNB-1	1430	5401	10784
SpFNB-2	1323	5355	10423
SpFNB-3	1294	5091	9762
SpFNS-1	1361	6579	11874
SpFNS-2	1523	5300	10930
SpFNS-3	1481	6481	12669
Total	2855	20460	49591

**Table 4:** DE miRNAs with targets between FNS and M03.

miRNA ID	Target	Annotation
*novel-m2307-5p*	*Isoform014322*	ENTH/ANTH/VHS superfamily protein isoform 1
*novel-m2430-5p*	*Isoform009669*	protein IQ-DOMAIN 14
*novel-m2524-5p*	*Isoform010547*	F-box and leucine-rich repeat protein 2/20
*novel-m2550-5p*	*Isoform007857*	defensin Ec-AMP-D2-like
*novel-m2554-3p*	*Isoform007343*	probable prefoldin subunit 2
*novel-m2572-3p*	*Spo15344*	plant mobile domain family protein
*novel-m2617-3p*	*Isoform008852*	uncharacterized LOC104898421
*novel-m2619-3p*	*Spo05571*	Polygalacturonase (PG) (3.2.1.15) (Pectinase) (Precursor)
*novel-m2641-5p*	*Isoform002028*	7-hydroxymethyl chlorophyll a reductase, chloroplastic
	*Isoform002329*	ABC transporter A family member 7-like
	*Isoform007233*	cysteine-rich and transmembrane domain-containing protein A
	*Isoform014680*	transcription factor bHLH90
*novel-m2763-3p*	*Isoform000331*	probable xyloglucan endotransglucosylase/hydrolase protein 5
	*Isoform011756*	uncharacterized LOC104897309

## Data Availability

Sequencing data that support the findings of this study has been deposited in the NCBI SRA database [https://www.ncbi.nlm.nih.gov/sra/PRJNA796451, accessed on 17 March 2022]. All data generated or analyzed during this study are included in this published article.

## Supplementary Materials

The following supporting information can be downloaded at: https://www.mdpi.com/article/10.3390/ijms23084090/s1,
