# Supplementary material for: Identification of Sex Differentiation-Related microRNAs in Spinach Female and Male Flower
Source: Int J Mol Sci. 2022 Apr 7;23(8):4090. doi: 10.3390/ijms23084090 (PMC9029227; doi:10.3390/ijms23084090)
Supplement: Supplementary file 1 [file ijms-23-04090-s001.zip › ijms-1643331-supplementary.pdf]

International Journal of *Molecular Sciences*

MDPI

Table S1. 74 DEG miRNAs between female and male flowers at three early developmental stages

ID	FNS_TPM	FNB_TPM	FYS_TPM	M03_TPM	M05_TPM	M10_TPM	Log_2(Fold Chang)			
							M03/FNS	M05/FNB	M10/FYS	
miR159-y	197451.47	165561.92	109662.72	31924.66	28332.55	26103.43	-2.63	-2.55	-2.07	female-biased
miR319-y	15511.00	21592.41	28685.43	7321.10	7098.91	5325.79	-1.08	-1.60	-2.43	female-biased
novel-m0291-3p	14.36	12.13	3.86	0.01	0.01	0.01	-10.49	-10.24	-8.59	female-specific
novel-m0297-5p	9.41	13.80	39.45	0.01	0.01	0.01	-9.88	-10.43	-11.95	female-specific
novel-m0541-5p	9.41	13.80	39.45	0.01	0.01	0.01	-9.88	-10.43	-11.95	female-specific
novel-m0629-3p	271.15	397.62	442.82	72.51	50.70	59.40	-1.90	-2.97	-2.90	female-biased
novel-m0710-3p	250.34	330.83	249.34	58.84	68.64	56.58	-2.09	-2.27	-2.14	female-biased
novel-m0731-5p	9.41	13.80	39.45	0.01	0.01	0.01	-9.88	-10.43	-11.95	female-specific
novel-m0789-5p	9.41	13.80	39.45	0.01	0.01	0.01	-9.88	-10.43	-11.95	female-specific
novel-m0790-5p	9.41	13.80	39.45	0.01	0.01	0.01	-9.88	-10.43	-11.95	female-specific
novel-m0883-3p	121.59	137.57	250.92	13.54	15.60	21.77	-3.17	-3.14	-3.53	female-biased
novel-m1168-5p	9.41	13.80	39.45	0.01	0.01	0.01	-9.88	-10.43	-11.95	female-specific
novel-m1648-3p	6.41	14.43	19.46	1.82	3.62	3.33	-1.81	-2.00	-2.55	female-biased
novel-m1704-3p	68.71	79.12	113.97	27.25	24.38	26.93	-1.33	-1.70	-2.08	female-biased
novel-m2073-5p	9.41	13.80	39.45	0.01	0.01	0.01	-9.88	-10.43	-11.95	female-specific
novel-m2222-3p	240.05	324.22	240.18	55.54	66.99	54.38	-2.11	-2.27	-2.14	female-biased
novel-m2263-5p	4.83	7.15	6.92	0.01	0.32	0.01	-8.92	-4.49	-9.44	female-biased
novel-m2418-5p	14.16	31.84	23.71	4.30	3.97	5.76	-1.72	-3.00	-2.04	female-biased
novel-m2774-5p	4612.80	5012.48	14506.13	736.48	813.61	592.34	-2.65	-2.62	-4.61	female-biased
novel-m2889-5p	9.41	13.80	39.45	0.01	0.01	0.01	-9.88	-10.43	-11.95	female-specific
miR156-x	6128.69	3142.56	2976.15	58340.41	30481.57	32347.35	3.25	3.28	3.44	male-biased
miR156-y	142.61	16.79	36.13	4351.10	3475.54	4543.62	4.93	7.69	6.97	male-biased
miR157-x	6041.50	3048.89	2928.64	58134.95	30363.59	32050.65	3.27	3.32	3.45	male-biased
miR157-y	126.42	4.33	18.70	4315.09	3453.46	4525.12	5.09	9.64	7.92	male-biased
miR3699-y	1.56	0.01	0.78	99.13	85.85	74.46	5.99	13.07	6.57	male-biased
novel-m0091-5p	51.48	56.01	60.33	3356.63	4262.34	8860.81	6.03	6.25	7.20	male-biased
novel-m0098-5p	2.92	0.42	0.01	74.04	54.51	26.65	4.67	7.03	11.38	male-biased
novel-m0116-5p	2.92	0.42	0.01	74.04	54.51	26.65	4.67	7.03	11.38	male-biased
novel-m0124-3p	0.01	0.01	0.01	5.30	2.09	2.99	9.05	7.71	8.23	male-specific

novel-m0160-5p	51.48	56.01	60.33	3356.63	4262.34	8860.81	6.03	6.25	7.20	male-biased
novel-m0201-5p	0.01	0.01	0.01	5.91	3.19	4.05	9.21	8.32	8.66	male-specific
novel-m0204-3p	0.01	0.01	0.01	5.38	3.61	2.78	9.07	8.49	8.12	male-specific
novel-m0298-3p	2.80	0.43	0.01	22.56	10.69	9.73	3.01	4.63	9.93	male-biased
novel-m0348-3p	0.33	0.01	0.73	32.88	25.01	17.95	6.65	11.29	4.63	male-biased
novel-m0348-5p	0.01	0.01	0.01	10.74	2.99	2.02	10.07	8.23	7.66	male-specific
novel-m0349-3p	0.33	0.01	0.73	32.88	25.01	17.95	6.65	11.29	4.63	male-biased
novel-m0349-5p	0.01	0.01	0.01	10.74	2.99	2.02	10.07	8.23	7.66	male-specific
novel-m0468-5p	0.65	0.01	0.01	21.29	25.07	19.63	5.03	11.29	10.94	male-biased
novel-m0575-3p	0.01	0.48	0.01	13.09	11.96	10.17	10.35	4.63	9.99	male-biased
novel-m0604-5p	0.01	0.01	0.01	13.61	16.99	8.60	10.41	10.73	9.75	male-specific
novel-m0634-5p	0.46	0.01	0.01	18.45	7.22	13.01	5.31	9.50	10.35	male-biased
novel-m0718-5p	0.01	0.01	0.01	8.95	17.26	11.33	9.81	10.75	10.15	male-specific
novel-m0869-5p	0.46	0.01	0.73	26.70	20.23	15.85	5.85	10.98	4.45	male-biased
novel-m0905-5p	0.65	0.01	0.01	21.29	25.07	19.63	5.03	11.29	10.94	male-biased
novel-m0916-5p	0.01	0.01	0.80	13.29	7.58	12.55	10.38	9.57	3.98	male-biased
novel-m0929-3p	9.42	4.94	0.78	105.41	55.39	57.08	3.48	3.49	6.19	male-biased
novel-m0991-3p	0.46	0.01	0.01	24.65	12.18	14.61	5.73	10.25	10.51	male-biased
novel-m1321-5p	51.48	56.01	60.33	3356.63	4262.34	8860.81	6.03	6.25	7.20	male-biased
novel-m1393-5p	0.01	0.01	0.01	7.90	11.67	9.35	9.63	10.19	9.87	male-specific
novel-m1455-3p	0.01	0.01	0.01	10.20	21.32	9.79	9.99	11.06	9.93	male-specific
novel-m1669-5p	0.01	0.01	0.01	13.61	16.99	8.60	10.41	10.73	9.75	male-specific
novel-m1700-5p	51.48	56.01	60.33	3356.63	4262.34	8860.81	6.03	6.25	7.20	male-biased
novel-m1701-5p	51.48	56.01	60.33	3356.63	4262.34	8860.81	6.03	6.25	7.20	male-biased
novel-m1785-3p	0.46	0.01	0.01	23.61	9.78	3.34	5.67	9.93	8.38	male-biased
novel-m1786-3p	9.42	4.94	0.78	105.41	55.39	57.08	3.48	3.49	6.19	male-biased
novel-m1805-5p	51.48	56.01	60.33	3356.63	4262.34	8860.81	6.03	6.25	7.20	male-biased
novel-m1876-5p	0.01	0.01	0.01	13.49	19.82	11.55	10.40	10.95	10.17	male-specific
novel-m1903-3p	9.42	4.94	0.78	105.41	55.39	57.08	3.48	3.49	6.19	male-biased
novel-m2154-5p	0.01	0.01	0.01	37.70	37.74	32.74	11.88	11.88	11.68	male-specific
novel-m2282-5p	0.01	0.01	0.01	11.91	5.56	6.02	10.22	9.12	9.23	male-specific
novel-m2640-5p	51.48	56.01	60.33	3356.63	4262.34	8860.81	6.03	6.25	7.20	male-biased
novel-m2689-3p	0.01	0.01	0.01	7.18	8.01	14.29	9.49	9.65	10.48	male-specific
novel-m2692-5p	2.39	0.43	4.61	203.38	234.25	240.21	6.41	9.08	5.70	male-biased

novel-m2882-3p	0.01	0.01	0.01	16.83	9.88	5.77	10.72	9.95	9.17	male-specific
novel-m2963-3p	0.01	0.01	0.01	10.20	21.32	9.79	9.99	11.06	9.93	male-specific
novel-m2996-3p	0.01	0.01	0.01	7.97	5.06	7.90	9.64	8.98	9.63	male-specific
novel-m3016-3p	9.42	4.94	0.78	105.41	55.39	57.08	3.48	3.49	6.19	male-biased
novel-m3034-3p	105.34	56.34	124.49	866.89	1327.37	754.97	3.04	4.56	2.60	male-biased
novel-m0392-3p	12.82	9.84	129.22	49.63	42.12	39.00	1.95	2.10	-1.73	
novel-m0426-5p	43.83	31.41	493.22	108.78	82.63	89.93	1.31	1.40	-2.46	
novel-m0519-3p	14.07	12.93	69.86	34.84	41.48	25.21	1.31	1.68	-1.47	
novel-m0675-5p	28.65	28.51	261.90	173.95	108.01	101.92	2.60	1.92	-1.36	
novel-m1957-5p	29.65	20.37	602.36	121.48	114.08	156.31	2.03	2.49	-1.95	
novel-m2917-5p	24.57	22.46	368.38	107.08	68.06	78.82	2.12	1.60	-2.22	

International Journal of
Molecular Sciences

MDPI

Table S2. 22 pairs of sex-biased miRNA-target

miRNA ID	Target ID	rho	Symbol	Description
miR156-x	Spo16283	-0.69	SPL6	Squamosa promoter-binding protein-like 6
miR157-x	Spo16283	-0.69	SPL6	Squamosa promoter-binding protein-like 6
miR159-y	Gene003818	-0.89	GAM1	transcription factor GAMYB-like
miR159-y	Gene004473	-0.88	GAM1	myb-like protein A
miR159-y	Spo11658	-0.76	--	BnaC01g19500D protein
miR159-y	Spo12050	-0.82	GAM1	myb domain protein 81
miR159-y	Spo22574	-0.86	--	Actin cytoskeleton-regulatory complex protein pan1, putative isoform 1
miR159-y	Spo25879	-0.77	HOS1	myb domain protein 65
miR319-y	Gene003818	-0.75	GAM1	transcription factor GAMYB-like
miR319-y	Spo12050	-0.83	GAM1	myb domain protein 81
miR3699-y	Spo16283	-0.70	SPL6	Squamosa promoter-binding protein-like 6
novel-m0298-3p	Spo13152	-0.82	--	Unknown protein
novel-m0348-3p	Gene001480	-0.76	Os01g0760900	probable protein ABIL5
novel-m0349-3p	Gene001480	-0.76	Os01g0760900	probable protein ABIL5
novel-m0883-3p	Spo08899	-0.85	At5g04160	Phosphate translocator-related family protein
novel-m1455-3p	Spo25368	-0.80	--	Unknown protein
novel-m1648-3p	Gene004165	-0.85	-	hypothetical protein CISIN_1g048346mg, partial [Citrus sinensis]
novel-m1785-3p	Gene003612	-0.79	-	E3 ubiquitin-protein ligase COP1-like; K10143 E3 ubiquitin-protein ligase RFWD2 [EC:2.3.2.27]
novel-m1876-5p	Spo06647	-0.73	ABCG11	ABC transporter G family member 11
novel-m2774-5p	Gene000991	-0.75	At4g10260	probable fructokinase-5; K00847 fructokinase [EC:2.7.1.4]
novel-m2774-5p	Spo09942	-0.80	At4g10260	Fructokinase
novel-m2963-3p	Spo25368	-0.80	--	Unknown protein

Table S3. DE miRNAs residing in sex chromosome

miRNA ID	$\log_2$(FC(M03/FNS))	miRNA ID	$\log_2$(FC(M05/FNB))	miRNA ID	$\log_2$(FC(M10/FYS))
novel-m2307-5p	9.15	miR1439-y	2.50	miR384-x	-2.05
novel-m2312-5p	3.47	novel-m2320-5p	-1.36	novel-m2312-5p	-8.86
novel-m2336-5p	8.27	novel-m2335-3p	14.28	novel-m2314-3p	-2.64
novel-m2358-5p	1.61	novel-m2337-5p	-8.76	novel-m2315-5p	-2.47
novel-m2360-3p	10.30	novel-m2341-5p	8.25	novel-m2320-5p	-1.56
novel-m2361-5p	-1.46	novel-m2351-3p	-2.00	novel-m2329-3p	-4.33
novel-m2362-3p	-1.01	novel-m2366-3p	7.62	novel-m2330-5p	-1.81
novel-m2389-5p	8.33	novel-m2384-3p	8.91	novel-m2331-5p	-2.63
novel-m2395-3p	7.85	novel-m2399-5p	-8.78	novel-m2333-3p	8.18
novel-m2412-3p	-1.06	novel-m2414-5p	3.39	novel-m2335-3p	6.75
novel-m2416-3p	-3.00	novel-m2417-5p	9.04	novel-m2336-5p	-4.32
novel-m2418-5p	-1.72	novel-m2418-5p	-3.00	novel-m2339-5p	-9.06
novel-m2420-3p	1.55	novel-m2419-5p	8.15	novel-m2342-5p	-1.93
novel-m2428-3p	7.68	novel-m2432-3p	3.69	novel-m2347-3p	-11.01
novel-m2430-5p	2.25	novel-m2445-5p	8.44	novel-m2351-3p	-2.99
novel-m2431-5p	1.06	novel-m2457-3p	1.71	novel-m2354-5p	-2.02
novel-m2443-5p	9.05	novel-m2462-5p	9.58	novel-m2357-3p	-2.52
novel-m2464-3p	-1.06	novel-m2463-5p	9.04	novel-m2371-5p	-3.93
novel-m2467-3p	1.94	novel-m2477-5p	8.67	novel-m2376-3p	-3.58
novel-m2469-3p	2.12	novel-m2483-5p	8.84	novel-m2384-3p	9.10
novel-m2472-5p	2.19	novel-m2500-5p	6.91	novel-m2396-5p	-1.45

novel-m2496-5p	2.37	novel-m2511-5p	8.22	novel-m2404-3p	-1.58
novel-m2498-3p	2.95	novel-m2514-5p	3.81	novel-m2405-5p	8.26
novel-m2506-5p	-7.85	novel-m2523-5p	8.21	novel-m2418-5p	-2.04
novel-m2508-3p	4.06	novel-m2525-3p	9.03	novel-m2426-3p	-4.70
novel-m2509-5p	8.58	novel-m2568-3p	-8.78	novel-m2430-5p	-2.05
novel-m2524-5p	7.51	novel-m2577-5p	1.54	novel-m2431-5p	-10.33
novel-m2535-3p	8.74	novel-m2583-5p	9.22	novel-m2432-3p	9.98
novel-m2543-5p	4.05	novel-m2585-5p	-8.78	novel-m2433-5p	-1.69
novel-m2550-5p	8.90	novel-m2593-5p	-1.26	novel-m2434-5p	-2.22
novel-m2554-3p	7.16	novel-m2599-3p	9.86	novel-m2435-5p	-3.67
novel-m2572-3p	9.26	novel-m2607-3p	7.74	novel-m2436-3p	-8.81
novel-m2574-3p	4.75	novel-m2608-3p	9.11	novel-m2437-5p	-3.80
novel-m2575-3p	4.08	novel-m2609-5p	-2.22	novel-m2440-5p	-11.41
novel-m2577-5p	2.53	novel-m2617-3p	7.71	novel-m2441-3p	-1.44
novel-m2580-5p	1.06	novel-m2617-5p	8.61	novel-m2444-5p	-9.80
novel-m2587-3p	4.06	novel-m2625-5p	-4.41	novel-m2451-5p	-1.81
novel-m2588-5p	2.26	novel-m2635-5p	7.25	novel-m2454-5p	-1.91
novel-m2595-3p	1.05	novel-m2640-5p	6.25	novel-m2457-3p	-1.83
novel-m2611-3p	1.99	novel-m2653-3p	-4.34	novel-m2459-5p	-9.93
novel-m2617-3p	8.27	novel-m2655-5p	3.81	novel-m2460-5p	9.19
novel-m2619-3p	-8.19	novel-m2673-3p	-9.00	novel-m2467-3p	-1.66
novel-m2629-3p	4.06	novel-m2681-5p	7.99	novel-m2468-3p	-2.77
novel-m2631-5p	9.05	novel-m2688-3p	3.91	novel-m2469-3p	-2.63
novel-m2634-5p	8.58	novel-m2689-3p	9.65	novel-m2472-5p	-1.33
novel-m2640-5p	6.03	novel-m2692-3p	14.28	novel-m2475-5p	-2.28
novel-m2641-5p	7.55	novel-m2692-5p	9.08	novel-m2476-5p	-2.28
novel-m2689-3p	9.49	novel-m2702-3p	-1.10	novel-m2487-3p	-4.53
novel-m2691-5p	1.11	novel-m2704-5p	3.81	novel-m2503-3p	-3.07
novel-m2692-5p	6.41	novel-m2712-3p	-1.63	novel-m2504-3p	-1.51
novel-m2700-5p	-8.66	novel-m2721-3p	-3.66	novel-m2504-5p	-10.83
novel-m2701-5p	8.14	novel-m2736-5p	-1.04	novel-m2507-3p	-10.02
novel-m2739-3p	8.46	novel-m2737-5p	8.39	novel-m2510-5p	-3.12
novel-m2758-3p	8.42	novel-m2741-5p	8.91	novel-m2512-3p	-1.77
novel-m2763-3p	8.33	novel-m2745-3p	-1.52	novel-m2516-3p	-3.97
		novel-m2750-3p	8.99	novel-m2520-3p	-10.06
		novel-m2756-3p	-9.00	novel-m2530-5p	-10.05
				novel-m2535-3p	7.57
				novel-m2538-5p	-2.45
				novel-m2539-5p	-2.48
				novel-m2543-5p	-2.77
				novel-m2545-3p	-1.72
				novel-m2546-5p	9.19
				novel-m2551-3p	-9.42
				novel-m2572-3p	-3.93
				novel-m2579-5p	-3.09
				novel-m2580-5p	-10.33
				novel-m2588-3p	-1.89
				novel-m2589-3p	-1.94
				novel-m2590-5p	-1.81
				novel-m2595-3p	-1.18
				novel-m2595-5p	-1.44

			novel-m2601-3p	-2.14
			novel-m2612-5p	-1.07
			novel-m2615-5p	-1.07
			novel-m2617-5p	-4.12
			novel-m2622-3p	-1.23
			novel-m2624-3p	-2.70
			novel-m2625-5p	-11.87
			novel-m2630-3p	-2.51
			novel-m2634-5p	8.40
			novel-m2638-5p	-1.81
			novel-m2639-5p	-1.65
			novel-m2640-5p	7.20
			novel-m2642-5p	-1.94
			novel-m2645-3p	-1.34
			novel-m2648-5p	-2.20
			novel-m2649-3p	-1.09
			novel-m2653-3p	-6.01
			novel-m2656-5p	-4.09
			novel-m2660-3p	-1.42
			novel-m2660-5p	-2.95
			novel-m2664-3p	-10.04
			novel-m2669-5p	-1.16
			novel-m2675-3p	-3.13
			novel-m2677-3p	8.72
			novel-m2679-5p	9.19
			novel-m2683-3p	-3.17
			novel-m2689-3p	10.48
			novel-m2692-3p	6.75
			novel-m2692-5p	5.70
			novel-m2699-3p	-1.09
			novel-m2702-3p	-3.50
			novel-m2706-3p	-1.85
			novel-m2712-3p	-10.16
			novel-m2715-3p	-9.20
			novel-m2718-5p	-5.41
			novel-m2722-5p	-2.14
			novel-m2734-5p	-2.44
			novel-m2739-3p	7.36
			novel-m2740-3p	-2.27
			novel-m2741-5p	9.10
			novel-m2745-3p	-1.96
			novel-m2755-5p	-1.81
**Total**	**55**	**57**	**114**	

Table S4. Primer sequence

	Primer name	Sequence (5'-3')
**qRT-PCR**	novel-m2307-5p-F1	CTGCAATTATCTCCATCACAA
	novel-m2430-5p-F	CCCGGAATACTCGCAATGGTTTG
	novel-m2524-5p-F	CCGGCATGAACTCACTATTTA
	novel-m2550-5p-F	TGCGGATCGAACACGGGTTA
	novel-m2554-3p-F	ATGGGACGGAGAGAGTATGA
	novel-m2572-3p-F	GCGTTCATGAACAACTCGTTTA
	novel-m2617-3p-F	CTATCTACGGAGGGACGACACTA
	novel-m2641-5p-F	ACTCCCTCCGTCCCTTAATGCTC
	novel-m2763-3p-F	GAACTGAACTGAACTGAACT
	novel-m2514-5p-F	CTCCCTCCGTTTCTTTTTGTTA
	novel-m2608-3p-F	TACTGACCTCGGGTCGCAAA
	novel-m2642-5p-F	TCAGCTTCTGATCTGATCAGC
	SpEF-F	ATCCTTCTTCTCTATCGTCCT
	SpEF-R	TAGTCCATCTCAGCTAGCCTC
	At5sRNA-Q-F	GATGCGATCATACCAGCACTAA
	At5sRNA-Q-R	GATGCAACACGAGGACTTCCC
**VIGS**	VIGS-miR172-F	GGAATTCGTTGTTTGCCGATGCGGTA
	VIGS-miR172-R	CGGGATCCTTTATTGCCGCTGCAGCAT
	VIGS-miR2550n-F	GGAATTCCAATTGTTGTTGCGGGGGATCGAA
	VIGS-miR2550n-R	CGGGATCCGTCGGATTAATGCGAGTCAAAGTC
**OE**	pre-miR172-F	GTTGTTTGCCGATGCGGTA
	pre-miR172-R	TTTATTGCCGCTGCAGCAT
	pre-miR2550n-F	CAATTGTTGTTGCGGGGGATCGAA
	pre-miR2550n-R	GTCGGATTAATGCGAGTCAAAGTC
